# Is There a Temporal Relationship between COVID-19 Infections among Prison Staff, Incarcerated Persons and the Larger Community in the United States?

**DOI:** 10.3390/ijerph18136873

**Published:** 2021-06-26

**Authors:** Danielle Wallace, John M. Eason, Jason Walker, Sherry Towers, Tony H. Grubesic, Jake R. Nelson

**Affiliations:** 1 Center for Violence Prevention and Community Solutions, School of Criminology and Criminal Justice, 411 N. Central Ave., Room 600, Phoenix, AZ 85004, USA; jwwalke4@asu.edu; 2 Department of Sociology, University of Wisconsin-Madison, Sewell Social Sciences, 1180 Observatory Dr., Madison, WI 53706, USA; jeason2@wisc.edu; 3 Institute For Advanced Sustainability Studies, Berliner Str. 130, 14467 Potsdam, Germany; TowersConsultingLLC@gmail.com; 4 Geoinformatics & Policy Analytics Laboratory, School of Information, University of Texas at Austin, Austin, TX 78712, USA; grubesic@utexas.edu (T.H.G.); Jake.Nelson@austin.utexas.edu (J.R.N.)

**Keywords:** COVID-19, incarceration, pandemic, incarcerated populations, correctional staff, prisons

## Abstract

Background: Our objective was to examine the temporal relationship between COVID-19 infections among prison staff, incarcerated individuals, and the general population in the county where the prison is located among federal prisons in the United States. Methods: We employed population-standardized regressions with fixed effects for prisons to predict the number of active cases of COVID-19 among incarcerated persons using data from the Federal Bureau of Prisons (BOP) for the months of March to December in 2020 for 63 prisons. Results: There is a significant relationship between the COVID-19 prevalence among staff, and through them, the larger community, and COVID-19 prevalence among incarcerated persons in the US federal prison system. When staff rates are low or at zero, COVID-19 incidence in the larger community continues to have an association with COVID-19 prevalence among incarcerated persons, suggesting possible pre-symptomatic and asymptomatic transmission by staff. Masking policies slightly reduced COVID-19 prevalence among incarcerated persons, though the association between infections among staff, the community, and incarcerated persons remained significant and strong. Conclusion: The relationship between COVID-19 infections among staff and incarcerated persons shows that staff is vital to infection control, and correctional administrators should also focus infection containment efforts on staff, in addition to incarcerated persons.

## 1. Introduction

In the United States, on 7 August 2020, a newly married couple held a 55-guest wedding reception in Millinocket, Maine, during the midst of the pandemic [1]. Weeks after, the reception had become a super spreader event, leading to over 177 infections. One wedding guest worked at York County Jail, who, after attending the reception, worked five consecutive 8-h shifts while showing symptoms of COVID-19 [1]. The staff member became one of the first confirmed COVID-19 cases at the jail, with the jail being tied to 82 subsequent cases among incarcerated persons, other correctional staff, and their family members [2].

Correctional institutions have long had experience dealing with infectious disease outbreaks in their populations. For instance, Lambert and colleagues (2016) estimate that between 2002 to 2013, the incidence of tuberculosis in prisons was between 3 to 37 cases per 100,000 incarcerated people (the general population rate was between three to five cases per 100,000) [3]. Among individuals in state prisons, there is an estimated infection rate of 38% for Hepatitis C [4,5]. That said, even with ample experience with infectious diseases, modern prisons have not dealt with a pandemic, nor with an infectious disease that is deadlier and more complex than tuberculosis or the Hepatitis variants commonly found in prisons, while simultaneously having a general lack of knowledge about the virus itself and how it behaves. 

The environmental and organizational conditions of prisons in the US may exacerbate the pandemic. Prisons in the US have been chronically short-staffed for years, including medical personnel [6], impacting the ability of prisons and correctional systems to adequately address the pandemic. For instance, in a recent report, the US Department of Justice (DOJ) suggested that staffing shortfalls were one of the biggest challenges for stopping the transmission of COVID-19 in prisons [7]. In the report, the DOJ went on to showcase a lack of staffing at Lompoc Federal Correctional Institution, suggesting that “the twin burdens of screening inmates and staff members for COVID-19 symptoms while still providing routine medical care to the institution’s approximately 2700 inmates” was a major challenge to tamping down outbreaks in prisons (p. 2). Just like understaffing, overcrowding in prisons is both common and represents another challenge for prisons and prison systems in controlling the pandemic. A lack of space in prison inhibits social distancing, cohorting prisoners (i.e., limiting movement in prison to smaller groups of prisoners at a time), creating areas for hand washing or sanitization, and medical isolation. In some prisons, solitary confinement rooms are being used as medical isolation spaces [8]. In the past, overcrowding in prisons has been ruled to violate constitutional rights [9] and created larger societal issues, like high levels of recidivism [10,11,12], mental health problems [13,14], and increased morbidity and mortality among current and formerly incarcerated populations [15,16,17,18].

The issues prisons face surrounding the pandemic and how to correct them, however, have been largely focused on prison conditions that impact the spread of COVID-19 among incarcerated populations. Many prisons and prison systems, including the federal Bureau of Prisons, have gone to extraordinary lengths to reduce the population of incarcerated individuals through either early release or home confinement to stop the spread of COVID-19 within prisons. Unfortunately, while focusing on incarcerated populations is necessary, it simultaneously ignores the potential that correctional staff has to influence infections both within and outside of prisons. What is more, the near-exclusive focus on prisoners as the primary carriers and transmitters of disease ignores the mechanisms that bring infectious diseases, including COVID-19, into the prison.

Prison staff represents a vector of disease transmission between the community and incarcerated individuals [19] because they are in the community for a large percentage of their time, piercing the membrane of the prison daily as they go to and from work [19,20]. As such, controlling COVID-19 infections in prison is a critical part of “flattening the curve” for both incarcerated persons and staff [19]. Moreover, because of their contact with both the prison and the community, correctional staff are likely an avenue for COVID-19 to enter the prison [20], whether at the beginning of a pandemic or during successive waves. In this work, we ask: is there a temporal relationship, statistically, between rates of COVID-19 infections among incarcerated persons and correctional staff, while accounting for COVID-19 infections in the general population surrounding a prison? To answer this question, we present statistical evidence of the relationship between incarcerated persons and staff infections in the Federal Bureau of Prisons (BOP) at the start of the pandemic in March to December 2020.

## 2. Materials and Methods

### 2.1. Data

Our data come from several sources, including the Federal Bureau of Prison’s (BOP) COVID-19 dashboard (see bop.gov/coronavirus, first accessed on 26 March 2020), which provides daily updates on active COVID-19 cases, recoveries, and associated deaths for staff and incarcerated persons by each prison in the federal prison system. We engaged in nightly, automated web scraping to collect this data, resulting in a longitudinal panel dataset of COVID-19 information across prisons. Occasionally (approximately 39 days out of the 279 in the study window), the BOP did not update their dashboard; when this occurred, we assumed flat growth in positive COVID-19 cases. Additionally, from the BOP, we obtained the weekly population of incarcerated persons and quarterly “prisoner-to-staff” ratios by prison (see https://www.bop.gov/about/statistics/statistics_staff_staffing_ratios.jsp, first access on 26 March 2020). County-level COVID-19 information and county population data for the study window came from John Hopkins University (see https://github.com/CSSEGISandData/COVID-19 for data and documentation, accessed 11 February 2021), where, since January 2020, the Center for Systems, Science and Engineering has maintained an online dashboard tracking COVID-19 information gathered from state and local public health authorities in the United States.

The BOP data used in this study include information on both staff and incarcerated persons from 134 facilities between 26 March 2020 and 31 December 2020, a window of time before vaccines for COVID-19 were available. The BOP is a federal prison system, established in 1930, designed to centralize the administration of prisons while providing safe, consistent, and humane care of those in their custody (see https://www.bop.gov/about/ for more details, accessed on 15 June 2020). The BOP system includes several different prison types, including those that are privately run, of minimal, low, medium, and high security, administrative detention centers (distinct from immigration detention centers), medical centers, and prison camps (see https://www.bop.gov/about/facilities/, accessed on 15 June 2020). In our study, a number of facilities were excluded from the analysis due to operational functions beyond incarceration, data reporting limitations, and natural disasters. First, 20 administrative facilities, which include both medical and transfer facilities, were excluded due to their unique, non-incarceration operational functions. Second, 12 privately run facilities were excluded given that these facilities do not report COVID-19 information for staff. Next, facilities within prison complexes, where there are multiple distinct prisons within one location, are often different in security level, incarcerated population levels, and staffing numbers; however, the BOP does not report prisoner-to-staff ratios for each facility in a prison complex, only the complex as a whole, preventing the calculation of the staff population. Given this missing data, 37 prisons within prison complexes were excluded. Third, two prisons, Estill Federal Correctional Institution (FCI) and Marianna FCI, were excluded from the analyses due to natural disasters which significantly damaged the facilities and changed their operational status (see https://www.bop.gov/about/statistics/statistics_staff_staffing_ratios.jsp, first accessed on 26 March 2020). In total, there were 63 prisons in the analysis. The prisons remaining in the analyses represent single-prison facilities at a location, which function like traditional prisons (i.e., not an administrative, medical, or detention center), and are of all security levels (i.e., minimum security to maximum security). 

Next, the prisons in this study are located in 27 states across the nation, including: Alabama, Arizona, California, Colorado, Connecticut, Florida, Georgia, Illinois, Kansas, Kentucky, Maryland, Michigan, Minnesota, New Hampshire, New Jersey, New York, Ohio, Oklahoma, Oregon, Pennsylvania, South Carolina, South Dakota, Tennessee, Texas, Virginia, Wisconsin, and Wyoming. The counties where the prisons were located have a variety of racial and ethnic compositions, with the average percent of the population in the county that is non-white is 30.9%, with a range of 4.1–87.9%. Additionally, the percent of the county population that is white is, on average, 69.1% (ranging from 12.1% to 95.9%). The percent of the county population that is African American is, on average, 12.9% (ranging from 1.1% to 82.9%). The percent of the county population that is Hispanic is, on average, 14.1% (ranging from 1.4% to 82.9%). Next, the levels of poverty in the counties that the prisons are located in also vary greatly, with the average percent of the population in poverty across counties being 17.6%, with a range from 6.4% to 41%. Finally, most of the prisons are situated in counties that are not predominately rural. The average percent rural is 42.3%, though the median is 35%; showing that over half of the prisons in the study are located in predominately urban areas. That said, six prisons are in counties reporting nearly 100% rurality.

### 2.2. Variables

**Figure 1:**
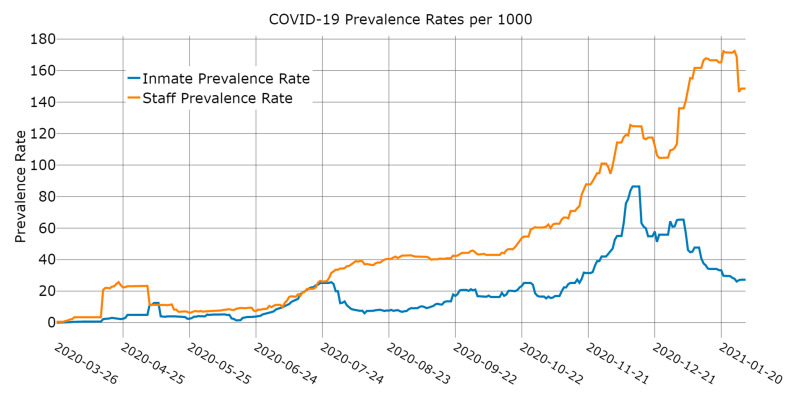
Prevalence rates of COVID-19 infections among staff and incarcerated persons.

Our dependent variable was the daily count of incarcerated individuals in a prison who tested positive and are still positive for COVID-19. Once the incarcerated person has recovered from COVID-19 or has died, they are moved out of the count of active positive cases (in Figure 1, this is displayed as a prevalence rate per 1000).

Our first independent variable was the rate of active COVID-19 per 1000 staff calculated by dividing the number of active COVID-19 cases among staff by the estimated current number of staff at the prison, then standardizing by 1000. We took the logarithm (+1) given its skewed distribution. The staff population was created by dividing the population of incarcerated persons by the prisoner-to-staff ratio (e.g., 18 prisoners to 1 staff). Our second independent variable was the incidence rate of COVID-19 cases per 1000 in the county where the prison sits, calculated by dividing the number of the total confirmed COVID-19 cases within the county by the estimated current county population. We then took the logarithm of the variable (+1) due to its skewed distribution. Note that the COVID-19 data for the county population represents the incidence of COVID-19 in the county surrounding the prison, whereas the COVID-19 data for incarcerated persons and staff represent individuals who have tested positive and are still positive or the prevalence of COVID-19 in these two groups.

We included several control variables. First, we included the lagged daily count of positive COVID-19 cases of incarcerated individuals in a prison. Next, the variable, homogenous policy, was a dummy variable signaling dates from 18 May 2020 to the end of the study window. The BOP changed a number of procedures surrounding home confinement, COVID-19 testing protocols, and the restriction of movements among detention centers between 8 May and 18 May 2020. Note that prior to 13 March 2020, the BOP had ceased all visitation, including visits with legal representation [21]; thus, the relationship between infections among staff and incarcerated persons is not confounded by other infection sources. Next, the BOP introduced a masking mandate for correctional officers on 27 August 2020 [22], regulating that all staff wore a mask when social distancing was not possible. This is accounted for through a dummy variable signaling the time period of where the mask mandate was in place (24 August 2020 to 31 December 2020). Additionally included is a linear time variable. Finally, we include fixed effects for each prison. Table 1 includes descriptive statistics for all variables, with the exception of the prison fixed effects.

### 2.3. Analysis Plan

We employed population-standardized regressions (i.e., negative binomial models with an exposure term) with fixed effects for prisons to predict the number of active cases of COVID-19 among incarcerated persons [23]. While several statistical specifications were considered to acknowledge the very likely presence of serial autocorrelation of our data, our ultimate model employed data that only included every fourteenth day of data in the model as a method to bypass serial autocorrelation. 

Several unique characteristics of the data informed this modeling strategy. First, to account for serial autocorrelation, the models are based on data from every *kth* day. We chose the value of *k* based on the infectious period of COVID-19 [24] and the inherently correlated nature of our outcome, given that it represents incarcerated persons who have tested positive for COVID-19 and remain positive. We use a value of *k* = 14, which falls outside of the median infectious period for COVID-19 of 13.4 days [24]. The resulting analytic dataset started on 3/27/20 and included 21 time points approximately 2 weeks apart nested within 63 prisons (n = 1323). We also examined other values of *k* following scholars who have established that the infectious window for asymptomatic spread is between 6.5 and 9.5 days, and between 10.9 and 15.8 days for symptomatic spread, with a median of 13.4 [24]. We estimated models based on values of *k* equal to 7, 10, 11 and 16; these models had similar results. We also tested whether the start day of the k-interval influenced the results using Hausman tests of stored estimates. Very few models showed significant differences; when this occurred, independent t-tests comparing individual coefficients with multiple comparison adjustments showed no true differences in estimates across models. Second, our outcome was significantly over the dispersed count variable, necessitating the use of non-linear modeling. Attempts to transform the outcome into a normally distributed variable failed given the functional form of log transformations and the large number of 0s and low counts in our outcome. Thus, generalized linear models using the negative binomial family were the most appropriate method of modeling the outcome. Third, and relatedly, when modeling the non-linear outcome, traditional statistical software also needed to account for autocorrelated panel data. The only known method for estimating a negative binomial panel model is through population average or generalized estimating equation [25] models with a first-order autocorrelated error structure; unfortunately, these models did not converge with traditional maximum likelihood algorithms. 

Given this, we employed a two-pronged approach to our analyses. First, we correct for correlated panel errors by using fixed effects for prisons. When specifying a fixed-effect model in negative binomial outcomes, Stata uses the conditional likelihood method to estimate fixed effects; unfortunately, this method does not fully control for unchanging covariates [26]. We addressed this by placing a dummy variable for each prison (i.e., a fixed effect) directly into the model [27,28]. Second, as explained earlier, we used data that only included every fourteenth day of data in the model as a method to circumvent serial autocorrelation. All analyses were conducted in Stata 15.

## 3. Results

Figure 1 shows the daily prevalence rates (per 1000) of COVID-19 among incarcerated persons and staff for the BOP prisons in our study. Keep in mind Figure 1 represents the aggregate trends in the data and may not be reflective of infection timelines in individual prisons. Upticks in the staff prevalence rate precede upticks to the prevalence rate for incarcerated persons, particularly during the first and third waves of COVID-19 infections in the data. Moreover, the staff prevalence rate is often starkly higher than the prevalence rate for incarcerated persons, particularly from mid-July 2020 through the end of the study window.

Table 2 shows the results from the negative binomial models (fixed effects for prisons are suppressed; see Appendix A). Model 1 included the main effects for the independent variables (i.e., the logged and lagged staff prevalence of COVID-19 and the logged and lagged incidence of COVID-19 in the county where the prison sits) and our control variables. We lagged the staff and county effects because if staff members have tested positive, they are not working in the prison; thus, any relationship between the prevalence of COVID-19 among staff and incarcerated persons is driven by past staff prevalence rates. The lagged and logged prevalence rate among staff had a significant relationship to COVID-19 infections among incarcerated persons: for each 1% increase in the staff prevalence rate, there is an associated 0.24% (95% CI: 0.135 to 0.342) increase in infections among incarcerated persons (note that because negative binomial models use a log link where the outcome is in the unit of logged events, employing logged predictors allows the estimated coefficient to be interpreted as an elasticity). Next, for every 1% increase in the lagged and logged incidence rate in the counties surrounding prisons, there is an associated 0.66% (95% CI: 0.294 to 1.018) increase in infections among incarcerated persons. Note that both of these coefficients is significantly larger than the coefficient for the lagged, daily count of COVID-19 infections among incarcerated persons (b = 0.006, 95% CI: 0.003 to 0.009); non-linear combination [29] tests (using “nlcom” in Stata) show that the coefficients are significantly different from one another.

To examine the implications of staff members cycling in and out of both the prison and the community on infections among incarcerated people, in Model 2, we moderated the logged and lagged staff prevalence of COVID-19 with the logged and lagged incidence of COVID-19 in the county population. Like the previous model, both variables had significant coefficients for predicting infections among incarcerated persons. Beyond these associations, the interaction coefficient between the lagged staff prevalence and the lagged county incidence rate is significant at *p* < 0.05 and negative (b = −0.24; 95% CI: −0.308 to −0.173). Additionally, in these models, the effect of the mask mandate is significant; during the mask mandate, there was an associated 0.84% (95% CI: −1.320 to −0.364) reduction in infections among incarcerated persons.

**Figure 2:**
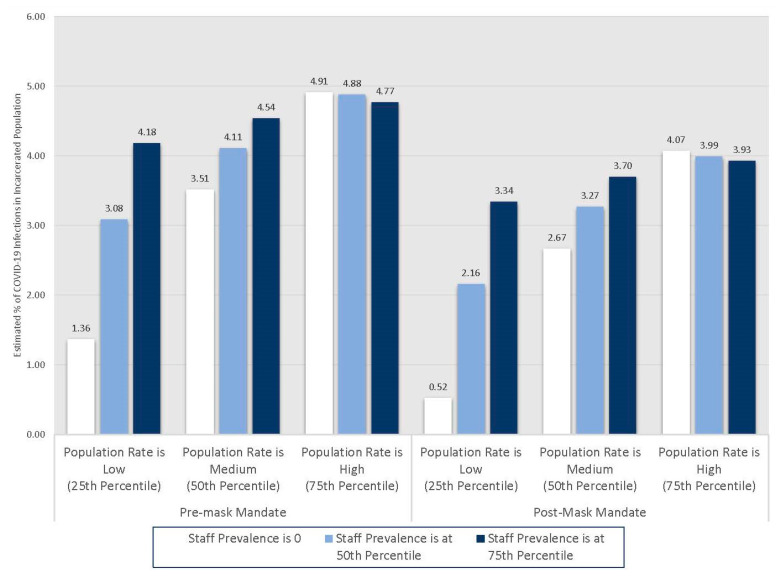
Point estimates for active cases of COVID-19 among incarcerated persons at the twenty-fifth, fiftieth, and seventy-fifth percentiles of the lagged and logged staff prevalence and county incidence of COVID-19 for pre- and post-mask mandate periods.

To better understand how the interaction shapes the relationships between these populations, we conducted a series of non-linear combination tests to examine whether the point estimates of both the staff prevalence rate and the county incidence rate at the twenty-fifth, fiftieth, and seventy-fifth percentiles for each variable and their interaction were not equal to 0. The distribution of the staff prevalence of COVID-19 is positively skewed, so much so that the twenty-fifth percentile is a value of 0 (non-zero rates begin to appear around the thirty-ninth percentile). Additionally, we also differentiate these results by the time periods before and after the mask mandate within the BOP. Figure 2 shows these results.

Beginning with the pre-mask mandate period, when the lagged and logged staff prevalence of COVID-19 is at 0 and the lagged, logged county population COVID-19 incidence rate is at the twenty-fifth percentile, there is an associated 1.36% (95% CI: 1.24–1.48) increase in active COVID-19 infections among incarcerated persons, net of controls. Next, when both the lagged and logged staff prevalence of COVID-19 and the lagged and logged county population COVID-19 incidence rate is at the median, there is an associated 4.11% (95% CI: 3.30–4.88) increase in COVID-19 infections among incarcerated persons, net of controls. Finally, when both the lagged and logged staff prevalence of COVID-19 and the lagged and logged county population COVID-19 incidence rate is at the seventy-fifth percentile, there is an associated 4.77% (95% CI: 3.66–5.88) increase in COVID-19 infections among incarcerated persons, net of controls. Of note is the influence the county incidence rate had on COVID-19 infections among incarcerated persons when the rate of staff infections is at 0. As the county incidence rate increased, even when the staff prevalence rate is 0, there was a significant positive effect on COVID-19 infections among incarcerated persons. Given that pathways for COVID-19 to enter prisons were significantly restricted with the exception of staff, these results are suggestive of asymptomatic spread by staff.

Turning to the mask mandate period, when the lagged and logged staff prevalence of COVID-19 is at 0 and the lagged, logged county population COVID-19 incidence rate is at the twenty-fifth percentile, there is an associated 0.52% (95% CI: 0.06–0.99) increase in active COVID-19 infections among incarcerated persons, net of controls. Next, when both the lagged and logged staff prevalence of COVID-19 and the lagged and logged county population COVID-19 incidence rate is at the median, there is an associated 3.27% (95% CI: 2.46–4.08) increase in COVID-19 infections among incarcerated persons, net of controls. Finally, when both the lagged and logged staff prevalence of COVID-19 and the lagged and logged county population COVID-19 incidence rate is at the seventy-fifth percentile, there is an associated 3.93% (95% CI: 2.81–5.05) increase in COVID-19 infections among incarcerated persons, net of controls. Note that while during the mask mandate period, infections among incarcerated persons were lower overall, the staff prevalence rate and the county incidence rates remain significantly associated with infections among incarcerated persons, perhaps suggesting continued asymptomatic spread and imperfect or inconsistent mask-wearing among staff. 

## 4. Discussion

The nexus between incarcerated persons, correctional staff, and the larger community is vital to understanding how to control infectious diseases and COVID-19 in prisons both in the United States and internationally. Many scholars have pointed to the importance of these potentially reciprocal dynamics [30] and hypothesized that correctional staff is an important node for transmission of COVID-19 to incarcerated persons [20,30,31,32]. In this study, we demonstrated that there is a significant relationship between the COVID-19 prevalence among correctional staff, and through them, the larger community, and COVID-19 prevalence among incarcerated persons in the US federal prison system. Moreover, even when staff rates are low or at zero, the interaction that staff members have with the larger community likely continues to facilitate the spread of COVID-19 to incarcerated persons via pre-symptomatic and asymptomatic transmission. Finally, the mask mandate was associated with only a small decline in infections among incarcerated persons; in short, even with strong infection control policies in place, correctional staff are associated with infection spread within prisons.

To keep their prison systems and their populations minimally impacted by the coronavirus pandemic, many states took drastic measures to eliminate or reduce COVID-19 infections within their prisons. Examples include the early release of incarcerated persons, especially those at high risk for serious complications from COVID-19, at-home confinement, halting all social or legal visitation of incarcerated persons, quarantining newly incarcerated persons, and increasing hand sanitizations [33,34,35,36,37]. Many policies prisons are using to mitigate the damaging impact of the pandemic downplay or ignore the hazard that staff present to keeping infections low in prisons. Policies for infection control within prisons and prison systems have been available for some time, and policy recommendations that are focused on, or at minimum, address staff members are common [20,31]. Specific policies related to controlling infections and their spread among staff include detailed and regularly maintained documentation of staff assignments and movements, medically furloughing staff when exposure occurs (when nonimmune or vaccinated), regular symptom screening for staff, and infection-control training [20,31].

Yet, many US prisons did not enact staff-specific policy for months, even though public health scholars have warned that staff members are likely nodes for bringing COVID-19 into prisons [20]. Masking policies are a prime example of this delayed response. Even though we have known since the early days of the pandemic that COVID-19 is most likely readily transmitted through aerosol particles and mask-wearing substantially reduces the risk of transmission [38,39], few correctional systems enforced mandatory mask-wearing among correctional staff. By mid-August, 2020, only 30 states required that state correctional staff wear a mask while working [40]. A few states have since adopted mandatory mask policies, such as South Dakota (25 September 2020) and Nevada (18 November 2020) [41,42], but around one-third of state correctional departments currently do not have mandatory mask-wearing policies for staff. 

In our study, the BOP had been strongly recommending mask usage as early as mid-April, but a mask mandate requiring the use of masks when social distancing was not an option was instituted on 27 August 2020 [22]. Our study demonstrated that the mask mandate for correctional staff was associated with a small (about 0.8%) reduction in the prevalence of COVID-19 among incarcerated persons, suggesting that a masking mandate was not highly effective at reducing COVID-19 prevalence among the incarcerated population in the BOP system. 

There are a few reasons for why this may be the case. The effectiveness of a policy is only as good as how the policy is written and then implemented. Discretionary components of policy, which was the case with the BOP’s mask mandate requiring mask-wearing when social distancing was not possible, allow for variation in implementation, potentially undermining the effectiveness of the policy. What constituted situations where social distancing was not possible was up to staff members to both determine and enforce. Moreover, the discretionary elements may have also enabled an organizational environment that was callous towards COVID-19 mitigation [43]. Through interviews with individuals incarcerated in high-security prisons, scholars have shown that some prison guards did not take infection control seriously, such as coming to work while sick or not consistently wearing gloves [43]. Organizational culture and acceptance of organizational deviance [44,45] (i.e., working while sick, acceptance of inappropriate ways to wear masks, or non-adherence to social distancing) in conjunction with a more discretionary masking policy may have undermined the purpose of the policy: to reduce infection spread. Similarly, since the start of the pandemic, mask-wearing in the US was highly political [46], with significant portions of American society against mask-wearing. Discretionary dimensions of masking policy may have also allowed for personal perspectives on mask-wearing to undermine the implementation of the policy. Regardless of the cause, when masks are not worn or worn effectively, a masking policy is unlike to stop the spread of infection, particularly the pre- or asymptomatic spread of COVID-19. Unfortunately, we are unable to determine within-prison masking practices and/or culture, which may serve to illuminate why the drop in the prevalence of COVID-19 among the incarcerated population during the mask mandate was small; future work in this area is needed. 

Our study has a few limitations of note. Within the US, prisons, such as other confined spaces such as nursing homes and factories, where there have been significant COVID-19 outbreaks, are not health surveillance systems and have faced difficulty establishing epidemiologically informed methods of collecting and reporting COVID-19 data [20]. Prison data systems are not designed to track the health information commonly needed to estimate the epidemiological metrics associated with tracking and understanding outbreaks and transmission dynamics. Additionally, as noted earlier, the BOP has data reporting practices that make system-wide analysis and internal comparisons difficult. The culmination of these issues results in a lack of transparency surrounding data and within-prison functions in the BOP system. Relatedly, the reporting of prevalence, not incidence rates, impacted our models. The metric of prevalence rates is more susceptible to issues of serial autocorrelation, given that many cases are captured across contiguous days. These data issues are common among prisons and prison systems [20] and should be addressed to facilitate the tracking and investigation of outbreaks and other infectious disease-related situations. Lastly, our modeling strategy was the best solution to modeling data that was complicated by serial autocorrelation (time and measurement), clustering (prisons), and low counts of the dependent variable (non-linear outcomes). When possible, models able to accommodate these issues simultaneously should be used in future research. 

## 5. Conclusions

For many infectious diseases, the mechanisms of spread among individuals are unique to how an environment both facilitates and inhibits person-to-person contact. How a virus spreads across individuals within the confines of a school, for instance, is likely different from how that same virus spreads through a prison. The two main groups of people in prisons—staff and those incarcerated—have different activities and person-to-person contact. For instance, in a minimum-security prison, incarcerated individuals may have a significant amount of contact with each other and a decent amount of contact with staff, whereas in a maximum-security prison, staff members have more contact with each other than they do with people who are incarcerated. Importantly, in both prison contexts, only staff members have contact with the community outside of the prison. Incarcerated individuals are purposefully isolated from society; thus, for those incarcerated to become infected with COVID-19, the virus needs to enter the prison. Staff members are perhaps the most important and most frequently ignored node of entry for COVID-19 in prisons. For instance, in the BOP, visitation of prisoners was halted almost immediately around the start of the pandemic, though a mask mandate for staff members was implemented in the summer of 2020. Staff exit and enter the prison for every shift, and when not at work, they are exposed to community levels of COVID-19. It is possible that many of the first COVID-19 cases among incarcerated individuals were driven by staff transmission and infections [20,31]. 

In this paper, while we do not estimate the causal, time-ordered process of how COVID-19 entered prisons in the federal system, we expose a very important aspect of understanding prison COVID-19 outbreaks: staff members’ COVID-19 prevalence, and through them, the larger community have a direct relationship on the prevalence of COVID-19 among incarcerated individuals. Our results lend strong support to the idea that staff members are an important node of infection transmission in prisons [20,31]. Next, while causal models would have been ideal, the strong, temporal association between the staff and incarcerated persons prevalence rates of COVID-19 suggest that developing policies and practices to mitigate how staff members spread COVID-19 within prisons is a critical element of outbreak control. Importantly, those policies should be in place and implemented at the start of the pandemic to stave off prison outbreaks, rather than mid-way through the pandemic, as the BOP did with their mask mandate in late August 2020.

## Figures and Tables

**Table 1:** Descriptive statistics.

	Mean	Median	Standard Deviation	Minimum	Maximum
Active positive COVID-19 cases among incarcerated persons	17.82	1.00	63.71	0	1039
Population of incarcerated persons	968.27	929.00	373.02	177	2762
Prevalence rate of staff COVID-19 infections (per 1000)	41.49	10.32	68.20	0.00	461.29
Incidence rate of COVID-19 cases in the county population (per 1000)	18.45	8.74	22.24	0.00	153.44
Homogenous policies, system-wide	0.81	-	-	0	1
mask mandate instituted (24 August 2020 and beyond)	0.52	-	-	0	1
Linear time in every fourteenth day	10.00	10.00	6.06	0	20

**Table 2:** Population standardized regressions with prison fixed effects predicting the active cases of COVID-19 among incarcerated persons (fixed effects suppressed; full models in Table A1, available in Appendix A).

	Model 1	Model 2
Lagged active positive COVID-19 cases among incarcerated persons	0.006 **	0.007 **
	(0.002)	(0.002)
Lagged and logged prevalence rate of staff COVID-19 infections (per 1000)	0.238 **	0.776 **
	(0.053)	(0.097)
Lagged and logged incidence rate of COVID-19 cases in the county population (per 1000)	0.656 **	1.158 **
	(0.185)	(0.194)
Lagged and logged rate of active staff COVID-19 cases per 1000 × lagged and logged rate of COVID-19 cases in the county population per 1000	-	−0.240 **
	-	(0.034)
Homogenous policies, system-wide	−0.607 **	−1.123 **
	(0.266)	(0.282)
Mask mandate instituted (24 August 2020 and beyond)	−0.469	−0.842 **
	(0.242)	(0.244)
Linear time in every fourteenth day	0.185 **	0.233 **
	(0.037)	(0.036)
Lnalpha	1.105 **	1.056 **
	(0.050)	(0.050)
Constant	−9.579 **	−10.156 **
	(0.621)	(0.648)
Observations	1260	1260

Standard errors in parentheses; ** *p* < 0.05.

## Data Availability

The data will be made publically available in late 2022, afer the grant supporting this study closes.

## Appendix A. Population Standardized Regressions with Prison Fixed Effects Predicting the Active Cases of COVID-19 among Incarcerated Persons

**Table A1:** Population standardized regressions with prison fixed effects predicting the active cases of COVID-19 among incarcerated persons.

	**Model 1**		**Model 2**	
	**B**	**SE**	**B**	**SE**
Lagged active positive COVID-19 cases among incarcerated persons	0.006 **	(0.002)	0.007 **	(0.002)
Lagged and logged prevalence rate of staff COVID-19 infections (per 1000)	0.238 **	(0.053)	0.776 **	(0.097)
Lagged and logged incidence rate of COVID-19 cases in the county population (per 1000)	0.656 **	(0.185)	1.158 **	(0.194)
Lagged and logged rate of active staff COVID-19 cases per 1000 × lagged and logged rate of COVID-19 cases in the county population per 1000			−0.240 **	(0.034)
Homogenous policies, system-wide	−0.607 **	(0.266)	−1.123 **	(0.282)
Mask mandate instituted (24 August 2020 and beyond)	−0.469	(0.242)	−0.842 **	(0.244)
Linear time in every fourteenth day	0.185 **	(0.037)	0.233 **	(0.036)
Aliceville FCI	0.541	(0.781)	0.511	(0.787)
Ashland FCI	0.774	(0.704)	0.625	(0.725)
Atlanta USP	0.793	(0.760)	0.370	(0.766)
Atwater USP	−0.063	(0.751)	−0.102	(0.765)
Bastrop FCI	0.692	(0.724)	0.823	(0.735)
Beckley FCI	1.097	(0.687)	0.764	(0.713)
Bennettsville FCI	0.810	(0.761)	0.852	(0.770)
Berlin FCI	0.716	(0.776)	1.173	(0.803)
Big Spring FCI	1.954 **	(0.714)	1.742 **	(0.730)
Byran FPC	−1.269	(0.830)	−0.830	(0.812)
Big Sandy USP	−0.202	(0.738)	−0.371	(0.764)
Canaan USP	0.965	(0.693)	0.748	(0.715)
Cumberland FCI	0.422	(0.737)	0.352	(0.759)
Danbury FCI	2.383 **	(0.786)	1.865 **	(0.802)
Duluth FPC	2.022 **	(0.703)	1.766 **	(0.732)
Dublin FCI	0.681	(0.708)	0.152	(0.737)
Edgefield FCI	0.673	(0.746)	0.385	(0.761)
Elkton FCI	3.139 **	(0.754)	2.600 **	(0.765)
Englewood FCI	1.197	(0.727)	1.188	(0.734)
El Reno FCI	0.667	(0.725)	0.766	(0.737)
Fairton FCI	1.900 **	(0.805)	1.659 **	(0.808)
Fort Dix FCI	1.518 **	(0.754)	1.186	(0.754)
Gilmer FCI	1.496 **	(0.694)	1.604 **	(0.727)
Greenville FCI	1.581 **	(0.708)	1.568 **	(0.726)
Herlong FCI	0.313	(0.749)	0.156	(0.762)
Jesup FCI	2.986 **	(0.707)	3.026 **	(0.717)
La Tuna FCI	0.666	(0.763)	1.150	(0.771)
Lee USP	−0.188	(0.729)	−0.276	(0.750)
Lewisburg USP	1.380	(0.707)	1.580 **	(0.720)
Loretto FCI	1.978 **	(0.700)	2.015 **	(0.718)
Leavenworth USP	1.035	(0.734)	0.997	(0.740)
Manchester FCI	1.797 **	(0.713)	1.605 **	(0.730)
Marion USP	2.164 **	(0.701)	2.199 **	(0.713)
McDowell FCI	0.189	(0.717)	0.052	(0.741)
McKean FCI	1.735 **	(0.690)	1.438 **	(0.721)
McCreary USP	0.257	(0.716)	0.097	(0.741)
Memphis FCI	0.475	(0.752)	0.478	(0.755)
Mendota FCI	−1.157	(0.799)	−1.283	(0.808)
Miami FCI	1.549	(0.795)	1.829 **	(0.805)
Milan FCI	1.934 **	(0.757)	1.455	(0.772)
Montgomery FPC	−0.554	(0.775)	−0.086	(0.770)
Morgantown FCI	0.685	(0.726)	0.534	(0.741)
Otisville FCI	0.984	(0.862)	0.691	(0.863)
Oxford FCI	2.052 **	(0.692)	1.906 **	(0.710)
Pekin FCI	1.255	(0.707)	1.330	(0.727)
Pensacola FPC	−2.740 **	(0.943)	−2.196 **	(0.936)
Phoenix FCI	0.448	(0.753)	0.420	(0.761)
Ray Brook FCI	1.818 **	(0.712)	1.742 **	(0.742)
Safford FCI	0.023	(0.759)	0.674	(0.766)
Schuylkill FCI	0.629	(0.722)	−0.014	(0.748)
Seagoville FCI	2.637 **	(0.750)	2.186 **	(0.758)
Sheridan FCI	0.234	(0.737)	0.328	(0.760)
Sandstone FCI	2.144 **	(0.686)	2.065 **	(0.699)
Tallahassee FCI	0.588	(0.753)	0.855	(0.759)
Talladega FCI	1.157	(0.743)	1.072	(0.751)
Texarkana FCI	0.415	(0.725)	0.588	(0.736)
Thomson USP	1.765 **	(0.698)	1.879 **	(0.712)
Terminal Island FCI	4.046 **	(0.757)	3.550 **	(0.765)
Three Rivers FCI	2.407 **	(0.730)	2.174 **	(0.735)
Waseca FCI	2.178 **	(0.715)	2.041 **	(0.728)
Williamsburg FCI	−1.032	(0.791)	−0.772	(0.787)
Yankton FPC	−0.685	(0.829)	0.527	(0.811)
lnalpha	1.105 **	(0.050)	1.056 **	(0.050)
Constant	−9.579 **	(0.621)	−10.156 **	(0.648)
Observations	1260		1260	

Standard errors in parentheses; ** *p* < 0.05.
